# Systemic Amyloidosis and Cardiac Autonomic Neuropathy Associated with Waldenstrom's Macroglobulinemia

**DOI:** 10.1155/2017/8795213

**Published:** 2017-06-13

**Authors:** Aasems Jacob, Rishi Raj, Warren Walkow

**Affiliations:** Department of Internal Medicine, Monmouth Medical Center, 300 2nd Ave, Long Branch, NJ 07740, USA

## Abstract

A 73-year-old male with long-standing Waldenstrom's macroglobulinemia complicated with systemic amyloidosis presented with a witnessed syncopal episode. He had complaints of orthostatic dizziness and palpitations for few months. Orthostatic hypotension and peripheral neuropathy were demonstrated on physical examination. EKG, 24-hour Holter monitoring, and 2D echocardiogram were unremarkable. MRI of the brain ruled out stroke. Patients with amyloidosis can develop cardiovascular disease through amyloid cardiomyopathy, small vessel disease, conduction defects, pericardial effusion, or autonomic denervation. After ruling out other life-threatening causes, Ewing's battery of tests was done to rule out cardiac autonomic neuropathy. Two heart rate tests and one blood pressure test were abnormal which indicated severe cardiac autonomic neuropathy. Cardiac autonomic neuropathy can mask symptoms of acute coronary syndrome and hence early diagnosis using the simple bedside maneuver is beneficial. The test is also important for prognostication. Absence of augmentation of cardiac output from inadequate autonomic stimulation will lead to postural hypotension, exercise intolerance, and tachycardia. There may be no change in heart rate with Valsalva or deep breathing both of which increase parasympathetic tone. As the condition progresses, it may result in cardiac denervation which can result in silent myocardial infarction, syncope, and sudden death.

## 1. Introduction

Waldenstrom's macroglobulinemia (WM) is a plasma cell dyscrasia manifesting as lymphoplasmacytic lymphoma in the bone marrow and monoclonal IgM gammopathy in the blood. It is a rare condition with a median age at diagnosis of 64 years and has male preponderance [1]. Amyloidosis can rarely occur along with WM and manifests with peripheral neuropathy, restrictive cardiomyopathy, nephrotic syndrome, hepatomegaly, and macroglossia. Neuropathy is a common complication of both WM and amyloidosis. While neuropathy in amyloid is from axonal demyelination, WM shows demyelination with more sensory involvement than motor. Syncope can manifest in patients with WM and amyloidosis in multiple ways. Hyperviscosity syndrome can result in stroke and cardiac infiltrative process may cause restrictive cardiomyopathy, with other reasons being arrhythmias and heart failure. Amyloidosis patients are also at high risk for bleeding with or without abnormal coagulation profile [2]. Autonomic nervous system can get affected resulting in orthostatic hypotension and syncopal episodes which are assessed using Ewing's battery of tests [3]. Here, we are evaluating the etiology of the syncopal episode in a patient with long-standing WM and systemic amyloidosis.

## 2. Case Presentation

73-year-old male came to the ED with a syncopal episode when he got up from the chair after eating lunch in a restaurant. A doctor at a nearby table, who came for help, could feel a regular pulse. He was made to lie supine with raised legs which improved his symptoms and he regained consciousness. There was no seizure activity, urinary incontinence, or choking on food. He did not have any residual weakness or confusion. Although patient did not have any previous episodes of syncope, he often had orthostatic dizziness and palpitations were present for few months. Most of his episodes were postprandial. His past medical history included Waldenstrom's macroglobulinemia diagnosed 21 years ago, complicated with systemic amyloidosis, peripheral neuropathy, and hypertension. He had treatment trials with rituximab, Cyclophosphamide, and prednisone despite which his disease progressed and he was on bortezomib for 2 months at the time of admission. The patient had recurrent pleural effusion without evidence of ascites and had Video-Assisted Thoracoscopic Surgery (VATS) decortications done 11 years ago. Progressive thickening of skin during that time prompted for a biopsy which revealed AL amyloidosis. Pulmonary function tests had shown restrictive lung disease and reduced gas transfer.

Physical examination in the emergency room revealed orthostatic hypotension. Blood pressure in supine position was 149/75 mm Hg (heart rate: 89/minute) and standing it was 118/70 mm Hg (heart rate: 73/minute). Heart rate was regular. His skin was taut and firm on palpation all over the body, especially his face. Palpation of neck, abdomen, and extremities were limited due to thickening of the skin. No focal neurological deficit was appreciated. Blood counts and differentials, electrolytes and metabolic panel, troponin-I, and BNP were normal. EKG showed normal sinus rhythm at rate of 62/min and low voltages with no acute ST-T wave changes. MRI brain ruled out stroke. 24-hour Holter monitoring showed normal sinus rhythm with a run of 6 beats of supraventricular tachycardia and occasional premature ventricular and atrial complexes. No symptoms were reported in the diary. Transthoracic echocardiogram was limited due to chest wall calcification. However, it showed grade 1 diastolic heart failure and no pericardial effusion. Ultrasound of bilateral carotid arteries revealed no significant stenosis. Patient's orthostatic hypotension was initially thought to be due to dehydration; however it did not improve with intravenous hydration. Ewing's battery of tests was done (Table 1).

Two out of the three heart rate tests yielded abnormal results. One blood pressure test was also abnormal. This had satisfied criteria for severe cardiac autonomic neuropathy (CAN) in our patient. Dose of his antihypertensive amlodipine was decreased to 5 mg from 10 mg. He was also given fludrocortisone 0.1 mg daily and noticed to have improvement in his symptoms on follow-up one month later. His blood pressure was stable on the medications. Treatment of underlying conditions including WM was continued as outpatient and patient was advised to take postural precautions.

## 3. Discussion

Monoclonal gammopathies can lead to the rare complication of AL amyloidosis in up to 2.2% of the patients [4]. However, it has only been sparingly documented in association with WM [5, 6]. Median time between diagnosis of both the conditions is 8 months [7]. 36% of these patients have cardiac involvement. Clinical findings of amyloid cardiomyopathy are found in 50 percent of patients with AL amyloidosis, while only under 5% of AA amyloidosis patients develop this [8, 9]. Senile amyloidosis (ATTR) can result in predominantly infiltrative cardiomyopathy. Some TTR gene mutations like Thr60Ala mutation or Appalachian amyloid can cause almost 100% cardiac involvement [10]. Amyloid cardiomyopathy usually manifests in the form of heart failure, small vessel disease, conduction system disease, pericardial effusion, or thromboembolism. The patients are at a very high risk of sudden cardiac death.

However, our patient had none of the above features on EKG or echocardiogram. Although mild speckled appearance was present in the ventricular wall and septum in the echocardiogram, normal left ventricular wall thickness and function ruled out infiltrative cardiomyopathy. CAN, most commonly associated with long-standing diabetes mellitus, is also described in association with amyloidosis, systemic lupus erythematosus, and rheumatoid arthritis [11]. Autonomic neuropathy has also been described in association with bortezomib therapy [12]. However, a temporal association could not be made in our case. Impairment in cardiac autonomic dysfunction is associated with silent coronary events, intraoperative cardiovascular complications, orthostatic hypotension, and increased mortality [13]. Absence of augmentation of cardiac output from inadequate autonomic stimulation will lead to postural hypotension, exercise intolerance, and tachycardia. There may be no change in heart rate with Valsalva or deep breathing both of which increase parasympathetic tone. As the condition progresses, it may result in cardiac denervation which can result in silent myocardial infarction, syncope, and sudden death [14]. Patients with postural hypotension commonly manifest it postprandially. Supine and standing blood pressure may fall profoundly after meals in autonomic neuropathy [15]. This is thought to be as a result of postprandial pooling of blood in splanchnic circulation and vasodilation from gastric peptides [16].

Diagnosis of CAN is achieved with the bedside testing through Ewing's battery of tests. The tests were devised for the objective diagnosis of diabetic cardiac neuropathy and also have prognostic significance [3]. Parasympathetic innervation to heart is mainly evaluated with the heart rate tests and sympathetic innervation with blood pressure tests although there is significant overlap. Based on the number of abnormal heart rate and blood pressure tests, disease severity is assessed [17]. Increase in mortality with CAN is secondary to masking of acute coronary events. Patients with postural hypotension should be advised to maintain hydration and take postural precautions with slow rising up from supine or sitting position. Use of elastic stockings and abdominal binders may be tried although it is poorly tolerated by patients. Avoiding large meals and meals rich in carbohydrates are effective in preventing postprandial hypotension. Pharmacological therapy includes fludrocortisone started at 0.1 mg daily. The dose can be increased up to 0.3 mg daily for clinical benefits. If patient remains symptomatic on first-line therapy alternate regimens including sympathomimetic agents like ephedrine, phenylephrine, or dextroamphetamine and midodrine may prove beneficial. A small number of patients will have symptoms despite all the therapies [18].

## Figures and Tables

**Table 1:** Ewing's battery of tests to assess cardiac autonomic neuropathy.

Test	Result	Normal	Borderline	Abnormal
Heart rate response to Valsalva maneuver (ratio of baseline to Valsalva)	1.17	≥1.21	1.11–1.20	≤1.10
Heart rate variation on deep breathing (expiration to inspiration in beats/minute)	8	≥15	11–14	≤10
Blood pressure response to sustained grip (before to after in mm of Hg)	17	≥16	11–15	≤10
Heart rate response from supine to standing position (ratio of R-R interval change in response to change in position from supine to standing)	0.72	≥1.04	1.01–1.03	≤1.00
Blood pressure response from supine to standing position (supine to standing in mm of Hg)	31	≤10	11–29	≥30
